# The “viral–metabolic” interaction mechanisms and management challenges in chronic hepatitis B combined with metabolic dysfunction-associated fatty liver disease: from clinical evidence to an integrated multidisciplinary framework

**DOI:** 10.3389/fimmu.2026.1920121

**Published:** 2026-08-20

**Authors:** Qianqian Zhu, Chengde Su, Qiaoqiao Yao, Ting Yang, Yali Xu, Mingdan Li, Qian Liu, Yawen Luo, Ping Yang

**Affiliations:** 1 Department of Nursing, Affiliated Hospital of Zunyi Medical University, Zunyi, China; 2 Department of Infectious Diseases, Affiliated Hospital of Zunyi Medical University, Zunyi, China; 3 School of Nursing, Zunyi Medical University, Zunyi, China

**Keywords:** “virus–metabolism” interaction mechanism, chronic hepatitis B, management, metabolic dysfunction-associated fatty liver disease, review

## Abstract

The comorbidity of chronic hepatitis B and metabolic dysfunction-associated fatty liver disease involves a bidirectional “virus–metabolism” interaction mechanism, presenting a clinical paradox characterized by improved virological markers alongside an increased risk of hepatocellular carcinoma. Therefore, this article aims to systematically review the interaction mechanisms and regulatory factors between the two conditions, and to propose an integrated “virus–metabolism” dual-axis management framework that includes metabolism-stratified antiviral therapy and synchronous metabolic intervention, so as to provide a theoretical basis for stratified management and behavioral intervention of the comorbidity.

## 1 Introduction

Chronic hepatitis B (CHB) and metabolic dysfunction-associated steatotic liver disease (MASLD) represent two major contributors to the global burden of liver disease. With the increasing prevalence of obesity, type 2 diabetes mellitus (T2DM), and changes in dietary patterns worldwide, the burden of MASLD is continuously rising. Epidemiological studies have shown that the prevalence of MASLD reaches approximately 70%, 75%, and 68.8% among individuals with overweight, obesity, and T2DM, respectively, with the prevalence among patients with T2DM in Eastern Europe reaching as high as 80.6% (1). Meanwhile, CHB remains one of the leading causes of liver-related morbidity and mortality worldwide, particularly in Asia and Africa. When these two highly prevalent liver diseases coexist in the same individual, the coexistence of viral infection and metabolic abnormalities has increasingly become an important clinical issue affecting long-term outcomes in patients with CHB. Previous studies have reported that hepatic steatosis occurs in approximately 32.8% of patients with CHB, with an even higher prevalence of 36.5% among Asian populations (2). Therefore, patients with CHB combined with MASLD may experience a dual pathological burden resulting from persistent viral infection and metabolic disturbances. Emerging evidence suggests that CHB and MASLD are not simply additive conditions but may involve bidirectional interactions between viral and metabolic pathways. For example, hepatitis B virus (HBV)-encoded X protein (HBx) has been shown to promote lipid accumulation in hepatocytes (3, 4), whereas a lipid-rich microenvironment may stabilize HBx expression and impair host immune surveillance (5). These mechanisms may contribute to an increased risk of hepatocellular carcinoma (HCC) among patients with CHB combined with MASLD (6, 7). However, some studies have also observed increased hepatitis B surface antigen (HBsAg) clearance or reduced HBsAg levels in patients with CHB combined with MASLD, suggesting a potential association with improved functional cure outcomes. Notably, this apparent improvement in virological markers does not necessarily translate into reduced clinical risk, as some patients continue to exhibit an elevated risk of HCC development, representing a clinical paradox in which improved viral biomarkers coexist with persistent disease progression risk.

Current single-disease management paradigms may not adequately address the long-term management needs of patients with CHB combined with MASLD. Further investigation into the underlying virus–metabolism interactions and their regulatory factors is therefore warranted. Current CHB management guidelines primarily focus on viral suppression, prevention of fibrosis progression, and reduction of HCC risk, whereas stratified management strategies for patients with concomitant metabolic abnormalities remain relatively limited (8, 9). Conversely, evidence supporting MASLD management strategies is largely derived from populations without chronic viral hepatitis, and their applicability to patients with CHB has not been fully established (10, 11). Consequently, clinical management of patients with CHB combined with MASLD remains challenged by the separation of antiviral management and metabolic intervention, highlighting the need to consider this condition as a complex liver disease entity with distinct pathophysiological characteristics and management requirements. In recent years, substantial progress has been made in understanding the coexistence of CHB and MASLD. Previous reviews have systematically summarized epidemiological characteristics, virus–metabolism interactions, and clinical outcomes associated with this condition (8, 12, 13), providing important insights into the complex relationship between viral infection and metabolic abnormalities. However, existing studies have largely focused on specific mechanisms or individual clinical questions, while systematic integration of how virus–metabolism interactions and immune regulation influence disease management strategies, long-term risk assessment, and modifiable factors remains insufficient. In particular, several aspects require further clarification, including the residual HCC risk under effective virological control, the multilevel factors influencing virus–metabolism balance, and the evidence stratification of different management approaches. Therefore, this review aims to establish a virus–metabolism dual-axis management framework and systematically summarize current evidence from multiple perspectives, including viral mechanisms, immune regulation, metabolic interactions, risk-modifying factors, and clinical management strategies, with the goal of providing a more comprehensive reference for risk assessment and long-term management of patients with CHB combined with MASLD.

## 2 Virus–metabolism interactions and clinical paradoxes in CHB combined with MASLD

### 2.1 Clinical characteristics of patients with CHB combined with MASLD

Patients with CHB combined with MASLD exhibit substantial clinical heterogeneity. The severity of hepatic steatosis shows a weakened correlation with body mass index (BMI), and significant intrahepatic lipid accumulation may also occur in individuals with normal body weight, suggesting that HBV infection may modify the conventional linear association between metabolic abnormalities and body weight (8).

This observation is consistent with the concept of lean MASLD, which refers to a condition characterized by hepatic steatosis despite a normal BMI. The disease progression risk and long-term outcomes of lean MASLD are comparable to those of non-lean MASLD, and its prevalence is relatively high in Asian populations, which also overlap geographically with regions of high HBV prevalence (14). These findings suggest that chronic HBV infection may represent a potential contributing factor to the development of lean MASLD.

Metabolic abnormalities in patients with CHB combined with MASLD also exhibit phenotypic heterogeneity. Simple hepatic steatosis has been associated with reduced viral load, whereas steatohepatitis accompanied by metabolic syndrome components may shift the predominant disease-driving factors from viral factors toward metabolic factors (10, 12). In addition, the interaction between viral infection and metabolic abnormalities appears to be stage-dependent. During the early stage of mild steatosis, metabolic alterations may suppress viral replication through innate immune activation; however, with progression to steatohepatitis, lipotoxicity-related stress responses may synergize with the pro-oncogenic effects of viral proteins and contribute to disease progression (15). Collectively, the nonlinear metabolic phenotype, heterogeneity of metabolic disturbances, and disease-stage-dependent characteristics collectively contribute to the complexity of managing patients with CHB combined with MASLD, in which bidirectional molecular interactions between viral and metabolic pathways represent an important pathological basis.

### 2.2 Mechanisms of virus–metabolism interaction and immune regulation in the progression of CHB combined with MASLD

#### 2.2.1 Virus-induced metabolic dysregulation

HBV can participate in hepatic lipid metabolic remodeling through multiple viral proteins, among which HBV X protein (HBx) is considered an important regulatory molecule involved in HBV-associated metabolic abnormalities. This mechanism has been repeatedly demonstrated in independent cellular experiments and rodent models. *In vitro* studies using hepatocyte-derived cell lines have shown that HBx can directly increase the expression of fatty acid transport protein 2 (FATP2) on the hepatocyte membrane. Through the FATP2–long-chain acyl-CoA synthetase 1 (ACSL1) and FATP2–peroxisome proliferator-activated receptor gamma (PPARγ) signaling pathways, HBx promotes long-chain fatty acid uptake and intracellular esterification, thereby facilitating triglyceride accumulation in hepatocytes (4, 16). In addition, HBx can inhibit the transcriptional activity of PPARα, reduce mitochondrial fatty acid β-oxidation, and further aggravate hepatic lipid accumulation (17, 18). Beyond the effects of HBx, cellular studies have demonstrated that excessive synthesis and endoplasmic reticulum retention of hepatitis B surface antigen (HBsAg) can induce the unfolded protein response and activate the sterol regulatory element-binding protein-1c (SREBP-1c) pathway, thereby promoting *de novo* lipogenesis in hepatocytes (19, 20).

In addition to directly regulating lipid metabolic pathways, HBV may indirectly disrupt hepatic lipid homeostasis through immunometabolic regulation. Functional *in vitro* experiments have demonstrated that HBV infection can suppress Toll-like receptor 4 (TLR4)-mediated innate immune responses (21, 22) thereby weakening the protective effects of TLR4 signaling against saturated fatty acid-induced lipotoxicity and promoting hepatic lipid accumulation (23). Furthermore, long-term antiviral therapy may be associated with metabolic abnormalities in some patients. A single-center retrospective clinical study reported that an antiviral treatment duration of more than five years was an independent risk factor for MASLD development among patients with CHB (24). The underlying mechanisms may involve potential effects of antiviral drugs on lipid metabolism as well as lifestyle changes during prolonged treatment periods, highlighting the importance of metabolic monitoring in patients receiving long-term antiviral therapy (25). Overall, HBV may contribute to hepatic lipid accumulation and the development of MASLD through multiple mechanisms, including direct regulation by viral proteins, immunometabolic dysregulation, and treatment-related metabolic alterations.

#### 2.2.2 Metabolic feedback regulation of HBV persistence

Multiple retrospective cohort studies and systematic reviews have consistently demonstrated that patients with CHB combined with moderate-to-severe MASLD exhibit lower serum HBsAg levels and higher rates of HBsAg seroclearance. This phenomenon has been consistently observed, particularly in Asian cohorts, and represents a relatively well-established clinical characteristic (6, 26). However, the molecular mechanisms underlying this observation have not been sufficiently validated in human liver tissues, and current explanations are mainly based on mechanistic hypotheses derived from *in vitro* studies. At the level of viral protein stability, *in vitro* cellular experiments have shown that saturated fatty acids can inhibit the ubiquitin–proteasome-mediated degradation of HBx, thereby prolonging its intracellular half-life and enhancing HBV transcriptional activity (27). Increased HBx stability may not only sustain viral transcription but may also promote immune evasion and persistent antigen stimulation, thereby aggravating host immune dysfunction. Consequently, persistent viral infection and metabolic inflammation may form a mutually reinforcing pathological state. At the level of viral assembly and secretion, cholesterol, as an essential component involved in viral envelope formation, may facilitate HBV particle assembly and secretion when abnormally accumulated within hepatocytes (28). Based on these *in vitro* findings, it has been proposed that even when some patients with CHB combined with moderate-to-severe hepatic steatosis exhibit reduced serum HBV DNA levels, active intrahepatic viral replication and viral particle assembly may still persist (29).

Beyond direct effects on HBV itself, metabolic remodeling may indirectly regulate HBV antigen expression and host antiviral immunity through reshaping the intrahepatic immune microenvironment. Cellular and animal studies suggest that persistent hepatic lipotoxic stress can activate TLR4 signaling in Kupffer cells. Importantly, the biological effects of TLR4 activation appear to be highly dependent on disease stage and the surrounding microenvironment. During early infection or transient activation, TLR4-mediated innate immune responses may enhance antiviral immunity and restrict HBV replication. However, under the chronic lipotoxic environment observed in patients with CHB combined with MASLD, sustained TLR4 activation may promote the polarization of Kupffer cells toward a pro-inflammatory phenotype, induce NLRP3 inflammasome activation, and increase the release of pro-inflammatory mediators such as IL-1**β** and TNF-**α**. These processes may contribute to the maintenance of chronic low-grade inflammation and further aggravate hepatic injury and immune dysfunction (15, 30). At the adaptive immune level, cellular and animal models have demonstrated that a lipid-rich microenvironment not only promotes regulatory T cell expansion and negatively regulates effector T cell function but also directly affects the metabolic fitness of CD8+T cells. Lipotoxic stress can impair mitochondrial function and glycolytic reprogramming, leading to reduced production of effector molecules and increased expression of immune checkpoint molecules, including PD-1 and TIM-3. These changes may ultimately result in metabolically induced T cell exhaustion and reduced functional antiviral immunity (31). Furthermore, *in vitro* transcriptional regulation experiments have shown that abnormal fatty acid activation mediated by long-chain acyl-CoA synthetase 1 (ACSL1) can alter histone acetylation patterns and suppress HBV S gene promoter activity, thereby reducing HBsAg expression (32). Therefore, the lower HBsAg levels observed in cross-sectional studies among patients with CHB combined with moderate-to-severe hepatic steatosis may reflect the combined effects of metabolic regulation of viral antigen expression and immune exhaustion, rather than indicating true functional clearance of HBV (33).

#### 2.2.3 Mechanistic basis of virus–metabolism synergy in hepatocarcinogenesis

Persistent virus–metabolism interactions may contribute to the remodeling of the hepatic microenvironment toward chronic inflammation, fibrosis, and a pro-tumorigenic state (34). At the metabolic level, studies using animal models and cellular systems suggest that lipotoxicity-induced oxidative stress and endoplasmic reticulum stress can continuously activate inflammation-related signaling pathways, including JNK and NF-κB. At the viral level, HBV-related proteins may further amplify innate immune inflammatory responses through TLR2/TLR4 signaling (35). Persistent activation of these pathways may contribute to the establishment of a pro-tumorigenic hepatic microenvironment and facilitate HCC development. Previous studies have demonstrated that HBV DNA integration into the host genome can induce insertional mutagenesis, chromosomal instability, and the formation of virus–host chimeric transcripts, representing important genetic mechanisms involved in HBV-associated hepatocarcinogenesis (36). Based on these findings from individual mechanistic pathways, it has been proposed that in patients with CHB combined with moderate-to-severe MASLD, metabolic abnormalities-induced oxidative stress may aggravate DNA damage and genomic instability, while HBV integration-related genetic alterations may further amplify chronic inflammatory signaling and abnormal cellular proliferation. Together, these processes may constitute a synergistic pathological basis for hepatocyte malignant transformation. Our previous meta-analysis demonstrated that patients with CHB combined with moderate-to-severe MASLD had a 1.77-fold increased risk of HCC development (6). This increased risk may be associated with enhanced genomic instability under lipotoxic conditions, persistent activation of JNK/NF-κB signaling, and CD8+T cell dysfunction (37). In addition, clinical observational studies have shown that patients with this comorbidity frequently exhibit dyslipidemia, particularly elevated small dense low-density lipoprotein cholesterol (sdLDL-C) and reduced high-density lipoprotein cholesterol (HDL-C). These metabolic abnormalities are not only associated with hepatic injury severity but may also indicate increased cardiovascular metabolic risk (38, 39). Therefore, CHB combined with moderate-to-severe MASLD should not be considered merely a coexistence of two independent diseases, but rather a complex high-risk condition involving the combined effects of persistent viral infection, lipotoxic injury, immune dysregulation, and metabolic abnormalities.

#### 2.2.4 A systems-level perspective on virus–metabolism–immune interactions in the progression of CHB with MASLD

Based on the aforementioned findings from individual mechanistic pathways, this review further proposes a systems-level integrative perspective of virus–metabolism–immune interactions. It should be emphasized that this framework represents a conceptual integration and interpretation of existing evidence rather than an established mechanistic model, and it requires further validation through comprehensive multi-omics studies and prospective clinical cohorts. Previous studies indicate that HBV infection, metabolic abnormalities, and immune dysregulation are not independent pathological processes but may form a dynamic regulatory network during disease progression. Persistent HBV infection can alter hepatocellular lipid homeostasis through viral protein-mediated metabolic reprogramming and influence host immune regulation (5, 18–23), Conversely, MASLD-related lipotoxicity and oxidative stress may affect viral protein stability, the intracellular environment supporting viral persistence, and antiviral immune responses (27–33). Therefore, disease progression in patients with CHB combined with MASLD is unlikely to be explained by a single pathogenic factor but may result from the integrated effects of persistent viral infection, metabolic abnormalities, and immune-mediated inflammation.

**Figure 1:**
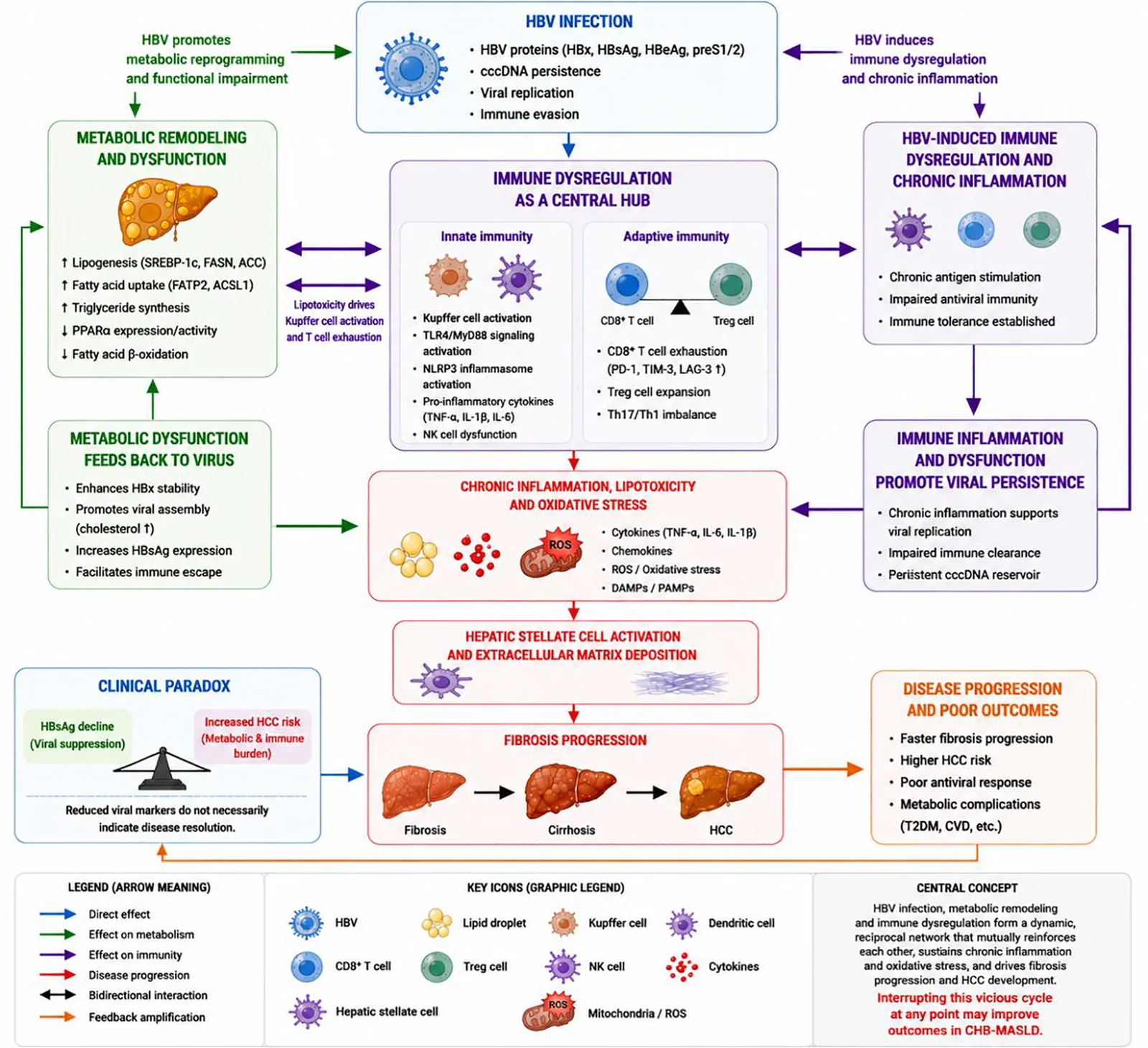
Schematic diagram of the bidirectional vicious cycle among HBV infection, metabolic dysfunction and immune imbalance driving liver fibrosis and hepatocellular carcinoma progression.

Within this network, immune responses serve as an important regulatory hub connecting viral stimulation and metabolic injury. Persistent HBV antigen exposure may induce T cell dysfunction and impaired antiviral immunity, whereas metabolic stress-derived lipotoxic signals may activate innate immune responses through inflammatory pathways such as TLR4, thereby promoting inflammatory microenvironment remodeling (23, 30, 31, 33–35). Thus, virus-associated immune dysregulation and metabolism-driven inflammatory responses do not occur independently but converge within the hepatic microenvironment to establish a persistent low-grade inflammatory state. With the continued activation of this pathological network, chronic inflammation, oxidative stress, and metabolic stress may collectively promote hepatic stellate cell activation and extracellular matrix deposition, making fibrosis a common pathological consequence of persistent viral infection, metabolic abnormalities, and immune-mediated inflammation (36, 39). Furthermore, the altered tissue microenvironment established during fibrosis progression may further sustain inflammatory activation and metabolic disturbances, thereby contributing to increased risks of cirrhosis and HCC development (36, 37, 40). Therefore, CHB combined with MASLD should be understood as a dynamic disease system jointly shaped by persistent viral infection, metabolic abnormalities, immune regulation, and fibrosis progression, rather than simply an overlap of two separate diseases. This systems-level understanding provides a theoretical basis for the subsequent virus–metabolism dual-axis dynamic risk management framework proposed in this review. Based on this mechanistic integration, we further developed a schematic representation of the virus–metabolism–immune–fibrosis dynamic regulatory network to summarize the interactions and feedback amplification processes among multiple pathological factors during the progression of CHB combined with MASLD (**Figure 1**).

### 2.3 Paradoxical clinical outcomes

The complex interaction between HBV infection and MASLD is reflected clinically by the coexistence of improved virological indicators and increased long-term disease risks. Several meta-analyses and pooled studies have demonstrated that patients with CHB with moderate-to-severe MASLD exhibit higher rates of HBsAg and HBeAg seroclearance than those with CHB alone; however, their risk of hepatocellular carcinoma (HCC) development may increase by approximately 1.77-fold (6). Currently, this phenomenon is considered not to represent true functional viral clearance, but may partly reflect suppressed viral antigen expression under conditions of metabolic dysregulation (26, 41). Meanwhile, lipotoxicity-associated inflammation, oxidative stress, and immune dysfunction may continue to promote hepatic fibrosis progression and hepatocarcinogenesis (40). These observations suggest that improvement in individual virological markers does not necessarily translate into long-term prognostic benefits in patients with CHB with MASLD. Therefore, clinical assessment should not rely solely on HBV serological responses, nor should patients be classified simply according to the presence or absence of hepatic steatosis. Instead, a comprehensive risk stratification approach incorporating the severity of steatosis, metabolic abnormalities, and hepatic fibrosis status may provide a more accurate assessment of disease progression and long-term outcomes.

### 2.4 Summary of evidence basis and limitations of key conclusions

Research in the field of CHB with MASLD encompasses multiple aspects, including molecular mechanisms, clinical characteristics, prognostic associations, and therapeutic exploration. However, the evidence supporting different conclusions varies substantially in terms of study design, evidence sources, and clinical applicability. To avoid interpreting findings from different evidence categories as equivalent, this review further summarizes the research basis, consistency of findings, and major limitations underlying the key conclusions (**Table 1**).

**Table 1:** Summary of research evidence supporting key conclusions in CHB with MASLD.

Conclusion category	Key conclusion	Evidence sources	Consistency across studies	Major limitations
Clinical epidemiology	MASLD is prevalent among patients with CHB and contributes to an increased overall hepatic disease burden	Multiple systematic reviews and large-scale cross-sectional studies	Highly consistent	Heterogeneity in MASLD diagnostic criteria among studies
Clinical outcomes	Moderate-to-severe MASLD is associated with an increased risk of HCC development in patients with CHB	Multiple cohort studies, systematic reviews, and meta-analyses	Generally consistent	Most studies have not completely excluded potential confounding factors, such as alcohol consumption and hepatitis C virus infection
Clinical outcomes	Patients with moderate-to-severe MASLD may exhibit greater reductions in HBsAg levels or higher rates of HBsAg seroclearance	Multiple retrospective cohort studies and individual meta-analysis	Relatively consistent	Most available data are derived from Asian populations, with limited direct evidence regarding intrahepatic viral replication
Molecular mechanisms	HBx promotes hepatic lipid accumulation through regulation of lipid metabolism-related pathways	Multiple *in vitro* cellular studies and animal models	Generally consistent	Most studies are based on hepatoma cell lines, and physiological relevance requires further validation in human tissues
Molecular mechanisms	A lipid-rich microenvironment may regulate HBV antigen expression and viral replication activity	*In vitro* cellular studies and small clinical studies	Partially consistent	Limited validation in human liver tissues, with discrepancies among different studies
Molecular mechanisms	Oxidative stress and HBV genomic integration may synergistically contribute to hepatocellular malignant transformation	Single-pathway mechanistic studies and theoretical integration	No consensus has been established	Direct validation in CHB with MASLD models is lacking, and causal relationships remain unclear
Modulatory factors	High-calorie and high-fructose dietary patterns contribute to MASLD progression	General population cohort studies and intervention studies	Highly consistent	Specific validation in populations with CHB with MASLD remains limited
Modulatory factors	Physical inactivity may aggravate metabolic abnormalities in patients with CHB with MASLD	Lifestyle intervention studies in MASLD populations and observational studies in CHB with MASLD populations	Generally consistent	Randomized controlled trials evaluating exercise interventions specifically in CHB with MASLD populations are lacking
Management strategies	Standard antiviral therapy combined with lifestyle intervention may improve clinical outcomes	Disease-specific guidelines and randomized controlled trials	Highly consistent	Available intervention evidence is mainly derived from single-disease populations, and applicability to CHB with MASLD requires further validation
Management strategies	A virus–metabolism dual-axis stratification framework may help optimize risk assessment and long-term management of CHB with MASLD	Conceptual integration based on mechanistic studies and observational evidence	No direct validation available	Prospective interventional studies specifically targeting CHB with MASLD populations are currently lacking

Overall, conclusions regarding clinical epidemiology and prognostic associations are supported by relatively large-scale clinical studies and therefore have a stronger clinical evidence foundation. In contrast, mechanistic conclusions are predominantly derived from *in vitro* cellular experiments and animal models, and their clinical translation remains to be further validated. Management and intervention-related conclusions are largely extrapolated from guidelines and evidence derived from individual diseases, with limited direct evidence specifically available for populations with CHB with MASLD.

## 3 Key factors regulating the “virus–metabolism” balance

At the molecular level, CHB and MASLD have established a bidirectional regulatory network with reciprocal interactions, while clinically exhibiting the paradoxical phenomenon of improved virological parameters coexisting with an increased risk of HCC. However, the virus–metabolism interaction is not a static process. Even among individuals with similar comorbid backgrounds, clinical outcomes remain highly heterogeneous. Therefore, beyond the intrinsic interactions between viral infection and metabolic abnormalities, multilevel factors including individual behavioral patterns, lifestyle exposures, and broader food environments may continuously modulate the dynamic virus–metabolism interplay and subsequently influence disease trajectories. Identifying these modifying factors and their potential pathways of action may provide a theoretical basis for integrated management and risk-stratified interventions in patients with CHB with MASLD.

### 3.1 Individual behavioral factors

#### 3.1.1 Dietary quality

Dietary quality is an important modifiable factor influencing hepatic lipid metabolic homeostasis and also represents a key exposure factor regulating the virus–metabolism interaction. Diets characterized by excessive caloric intake, high saturated fatty acid content, and high fructose consumption may promote hepatic *de novo* lipogenesis through activation of the SREBP-1c and ChREBP pathways, thereby exacerbating abnormal intrahepatic triglyceride accumulation. Meanwhile, these dietary patterns may further aggravate hepatic inflammation through activation of JNK/NF-κB signaling pathways (42, 43). In the context of CHB infection, diet-induced lipotoxicity may not only directly disrupt metabolic homeostasis but also indirectly modulate viral activity by affecting the stability of HBV replication-related proteins and viral assembly processes. This suggests a potential synergistic interaction among dietary exposure, metabolic dysfunction, and viral persistence, thereby contributing to disease progression (28). Furthermore, changes in modern dietary patterns have further amplified the risk of CHB with MASLD. Globally, the increasing consumption of ultra-processed foods has become an important contributor to the growing burden of MASLD (44). These foods are characterized by high energy density, increased proportions of saturated fatty acids and refined carbohydrates, as well as various industrial additives. Such components may simultaneously aggravate hepatic injury through metabolic and inflammatory pathways (45). Studies have demonstrated significant differences among populations and regions in dietary patterns, food accessibility, and cultural preferences, which may further influence the level of MASLD-related risk exposure (46). Hispanic populations and other minority groups may experience a higher burden of MASLD partly due to greater consumption of ultra-processed foods. Considering dietary characteristics and rapid nutritional transitions in the Chinese population, consumption of takeout foods and packaged foods has increased substantially, accompanied by a gradual rise in unhealthy dietary patterns characterized by excessive fat and sugar intake. Previous studies have suggested that these dietary patterns may promote intrahepatic lipid accumulation by disrupting gut microbiota homeostasis, thereby increasing the risk of MASLD development among patients with CHB (47, 48). In summary, dietary factors may influence the progression of CHB with MASLD through population-specific pathways, and future dietary intervention strategies should consider differences in dietary patterns and metabolic characteristics among individuals to facilitate more personalized management approaches.

#### 3.1.2 Physical activity and sedentary behavior

Physical activity is an important modifiable factor for maintaining metabolic homeostasis and may indirectly influence the dynamic balance between viral and metabolic pathways. Previous studies have demonstrated that exercise may exert beneficial effects on metabolic regulation by improving insulin sensitivity, enhancing fatty acid oxidation in skeletal muscle, and suppressing hepatic inflammatory signaling pathways. Evidence suggests that moderate-intensity aerobic exercise can reduce intrahepatic diacylglycerol accumulation and improve hepatic insulin signaling, with these effects occurring independently of weight reduction (49). In contrast, sedentary behavior is independently associated with increased hepatic fat accumulation and aggravated insulin resistance. By worsening the metabolic microenvironment, prolonged sedentary behavior may contribute to the shift of virus–metabolism interactions toward a pathological state (50). In patients with CHB with MASLD, insufficient physical activity may not only further disrupt metabolic homeostasis but also potentially impair host antiviral immune responses, thereby increasing the risk of adverse long-term outcomes. However, patients with CHB and hepatic steatosis are predominantly middle-aged and older males (51), a population that commonly exhibits sedentary lifestyles and relatively limited exercise tolerance. Therefore, exercise interventions should be tailored according to individual characteristics, including age, cardiopulmonary function, and severity of liver injury, with low-intensity and progressive exercise programs considered to improve long-term adherence (52).

#### 3.1.3 Other lifestyle-related factors

Beyond diet and physical activity, other lifestyle-related factors, including sleep patterns and psychological status, may also influence the virus–metabolism interaction through their effects on metabolic homeostasis. Studies have suggested that sleep disruption and circadian rhythm disturbances may alter hepatic lipid metabolism through impaired melatonin signaling and gut microbiota dysregulation, thereby increasing the risk of MASLD progression (53). Psychological stress may promote hepatic gluconeogenesis and lipolysis through excessive activation of the hypothalamic–pituitary–adrenal axis and sympathetic nervous system, thereby increasing metabolic stress on the liver (54). Furthermore, medication adherence among patients receiving long-term antiviral therapy represents another important factor influencing the virus–metabolism balance. Poor adherence may result in viral rebound and potentially aggravate metabolic abnormalities by disrupting the hepatic metabolic microenvironment, thereby contributing to a vicious cycle of “viral rebound–metabolic disturbance.” Therefore, comprehensive intervention strategies should also incorporate attention to sleep management, psychological regulation, and optimization of long-term treatment adherence.

### 3.2 Macro-environmental factors

#### 3.2.1 Food insecurity

Food insecurity refers to a condition in which individuals are unable to consistently access sufficient quantities of nutritionally adequate foods due to economic or social constraints (55). Recent studies have demonstrated that food insecurity is closely associated with a higher prevalence of MASLD, accelerated liver fibrosis progression, and increased all-cause mortality (56). Food insecurity may force individuals to select inexpensive foods with high energy density but poor nutritional quality. This constrained dietary pattern may further aggravate hepatic lipotoxicity and oxidative stress, disrupt metabolic homeostasis, and subsequently disturb the virus–metabolism balance, thereby contributing to adverse hepatic outcomes. In addition, food insecurity exhibits clear social gradients, with individuals living in low-income communities and minority populations often experiencing a greater burden of food insecurity. Such structural disparities represent important upstream determinants of health inequities in metabolic liver diseases and may partly explain population-level differences in clinical outcomes among patients with CHB with MASLD (57). Therefore, when developing management strategies for this comorbid condition, it is important to consider not only standardized dietary recommendations but also patients’ food accessibility and economic constraints. Otherwise, even well-designed nutritional interventions may have limited effectiveness in vulnerable populations due to real-world barriers to obtaining appropriate foods.

#### 3.2.2 Food system transitions

The ongoing structural transformation of global food systems has increased metabolic risks among individuals with liver diseases and may influence the virus–metabolism balance at the population level. A multinational study demonstrated that the expansion of ultra-processed food retail markets, changes in food pricing systems, and dietary transitions toward higher energy density and lower dietary fiber intake were significantly associated with increased MASLD prevalence and liver-related mortality. Moreover, these associations varied across countries with different levels of socioeconomic development (44). In China, rapid urbanization and food industrialization have gradually altered traditional dietary patterns. The widespread availability of takeout foods, packaged foods, and highly processed foods has increasingly exposed individuals with chronic liver diseases to a food environment that may promote metabolic dysfunction (58). Such environmental exposures may not only increase the risk of obesity, insulin resistance, and lipid metabolic disorders but also potentially impair the ability of the host to maintain virus–metabolism homeostasis. Meanwhile, macro-level food system transitions and individual-level food insecurity may interact and jointly create multilevel risks extending from social structures to health behaviors, thereby continuously amplifying the pathological burden of CHB with MASLD. Therefore, management of this comorbid condition may require not only interventions targeting individual lifestyles but also incorporation of food environment optimization and social support systems into an integrated management framework. Such approaches may help improve the broader regulatory context underlying the virus–metabolism dual-axis balance at the population level.

## 4 Construction of a virus–metabolism dual-axis management framework for CHB with MASLD

### 4.1 Current guideline-based management approaches and challenges in their application to patients with CHB with MASLD

The management of MASLD and metabolic disorders has gradually shifted from a single-specialty approach toward multidisciplinary collaboration. In 2025, the American Diabetes Association recommended routine screening for MASLD and hepatic fibrosis risk stratification among individuals with prediabetes or type 2 diabetes mellitus (59). Similarly, the Chinese *Primary Care Guidelines for the Diagnosis and Management of Metabolic Dysfunction-Associated Steatotic Liver Disease (2025)* emphasized the importance of multidisciplinary management approaches (60). In clinical practice, the concept of integrated management for metabolic liver diseases has gradually emerged, and some medical centers have established multidisciplinary clinics involving hepatologists, nutrition specialists, and weight management teams, providing a practical foundation for comprehensive MASLD management. However, systematic integration of long-term metabolic risk assessment and comprehensive management strategies for patients with concurrent metabolic abnormalities remains limited. In contrast, CHB-related guidelines primarily focus on controlling viral replication, preventing fibrosis progression, and reducing HCC risk, with relatively limited consideration of long-term metabolic risk management in patients with coexisting metabolic abnormalities. Therefore, although current CHB and MASLD guidelines have established respective frameworks for antiviral control and metabolic intervention, an integrated pathway addressing virus–metabolism interactions and dynamic risk management in patients with both conditions remains lacking. In current clinical practice, the management of patients with CHB with MASLD is still largely based on integrating recommendations from single-disease guidelines (61). During antiviral therapy, changes in body weight, glucose and lipid metabolism abnormalities, and progression of hepatic steatosis are often insufficiently considered, while lifestyle interventions are rarely tailored according to individual virological characteristics (62). In addition, nursing assessments predominantly focus on virological parameters and medication adherence, whereas dynamic monitoring of metabolic indicators, including body weight, waist circumference, dietary patterns, and physical activity levels, remains relatively insufficient. This may result in delayed identification of high-risk patients and potentially contribute to increased risks of hepatic fibrosis progression and HCC development (63). Therefore, the major challenge in managing CHB with MASLD does not simply involve combining antiviral therapy with metabolic interventions, but rather establishing an integrated management framework incorporating viral control, metabolic regulation, and immune-related risk assessment. Based on this concept, we propose a virus–metabolism dual-axis dynamic risk management framework that incorporates virological status, metabolic abnormalities, and long-term disease risks into a unified assessment system. This framework aims to facilitate a transition from a single-disease control model toward a comorbidity-oriented dynamic risk management approach and further clarify its potential applicability and clinical value through systematic comparison with current CHB and MASLD management strategies (**Table 2**).

**Table 2:** Comparison of the virus–metabolism dual-axis dynamic risk management framework with current CHB and MASLD management strategies.

Dimension	CHB management strategies	MASLD management strategies	Virus–metabolism dual-axis dynamic risk management framework
Disease management concept	Primarily focuses on persistent HBV infection, viral replication control, and host immune responses, with emphasis on hepatitis activity, fibrosis progression, and HCC risk	Primarily focuses on metabolic dysfunction, lipotoxicity, and metabolic inflammation, with emphasis on steatosis progression and fibrosis risk	Conceptualizes CHB-MASLD as a comorbid condition driven by interactions between viral factors and metabolic dysfunction, highlighting the interplay between virus-associated immune responses and metabolic inflammation
Pathophysiological focus	Focuses on HBV replication, immune dysregulation, and chronic inflammatory processes	Focuses on insulin resistance, lipotoxicity, oxidative stress, and metabolic inflammation	Integrating HBV-related immune dysregulation with MASLD-associated metabolic inflammation provides a mechanistic explanation for the persistent disease progression observed in the comorbid state.
Risk assessment and dynamic monitoring	Primarily based on HBV DNA levels, ALT, HBeAg/HBsAg status, fibrosis stage, and conventional HCC risk factors	Primarily based on metabolic abnormalities, severity of steatosis, and fibrosis risk assessment	Integrating virological parameters, metabolic status, fibrosis progression, and HCC-related risk factors, together with insights from immune-inflammatory mechanisms, enables dynamic risk stratification and long-term monitoring.
Therapeutic decision-making	Antiviral treatment decisions are mainly guided by viral replication status, hepatic inflammation, and disease stage	Management mainly relies on lifestyle modification and metabolic-targeted interventions	Integrates metabolic risk assessment into long-term antiviral management and promotes coordinated antiviral and metabolic interventions
HCC risk management	HCC surveillance is guided by HBV-related virological characteristics, fibrosis severity, and clinical risk factors	HCC risk assessment is mainly based on MASLD-associated fibrosis severity and metabolic risk factors	Incorporates the combined effects of persistent viral infection and metabolic abnormalities to enable individualized and dynamic HCC risk management
Long-term management model	A virus-centered, single-disease management approach focusing on HBV control	A metabolism-centered, single-disease management approach focusing on metabolic improvement	Proposes a dynamic virus–metabolism dual-axis management model

### 4.2 Virus–metabolism dual-axis management framework

Current CHB and MASLD guidelines primarily provide recommendations targeting viral control and metabolic abnormality management separately. However, an integrated risk stratification strategy that simultaneously considers viral activity and the severity of metabolic abnormalities remains insufficiently established. In this review, we propose a two-dimensional risk stratification framework based on viral activity and metabolic abnormality severity, integrating intervention and monitoring strategies with different levels of supporting evidence to provide a potential clinical decision-making framework. Within this framework, virological status is stratified according to HBV DNA levels, HBsAg/HBeAg status, and hepatic fibrosis severity. Low viral activity is defined as HBV DNA <2000 IU/mL, HBsAg positivity with absent or mild hepatic fibrosis (FIB-4 <1.30 or liver stiffness measurement [LSM] <8 kPa), whereas high viral activity is defined as HBV DNA ≥NAin IU/mL, HBeAg positivity or high HBsAg levels, accompanied by moderate-to-severe hepatic fibrosis (FIB-4 ≥FIB- or LSM ≥S kPa) (11, 64, 65). Metabolic status is stratified according to BMI, glucose and lipid metabolism abnormalities, and imaging-based assessment of hepatic steatosis. Mild metabolic abnormality is defined as BMI <24 kg/m², absence of diabetes mellitus or dyslipidemia, and mild steatosis detected by imaging. Moderate-to-severe metabolic abnormality is defined as BMI ≥24 kg/m², or the presence of type 2 diabetes mellitus, dyslipidemia, hypertension, or moderate-to-severe hepatic steatosis identified by imaging (11, 65). Based on this two-dimensional stratification, patients are dynamically categorized into four risk quadrants (**Table 3**), with corresponding management strategies and monitoring approaches assigned to each category.

**Table 3:** Virus–metabolism dual-axis dynamic risk stratification and management matrix for CHB with MASLD.

Metabolic status/viral status	Low viral activity	High viral activity
Mild metabolic abnormality	Quadrant I: Routine monitoring Viral axis: Antiviral therapy^①^ Metabolic axis: Basic lifestyle intervention^①^ Monitoring: Comprehensive assessment of virological and metabolic parameters may be considered every 6–12 months^③^	Quadrant II: Intensified viral control Viral axis: Antiviral therapy^①^ Metabolic axis: Lifestyle intervention^③^ Monitoring: Combined virological and metabolic assessment every 3–6 months^③^; initiation of dynamic fibrosis monitoring using FIB-4 and LSM^②^
Moderate-to-severe metabolic abnormality	Quadrant III: Intensified metabolic intervention Viral axis: Antiviral therapy^①^ Metabolic axis: Metabolic medications including GLP-1 receptor agonists, SGLT-2 inhibitors, and statins^①^; intensive lifestyle intervention with 5–7% weight reduction (supported by clinical evidence) Monitoring: Metabolic and fibrosis assessment every 3–6 months^③^	Quadrant IV: Intensified dual-axis management Viral axis: Antiviral therapy, with consideration of agents having fewer potential metabolic effects^①^ Metabolic axis: Combination of metabolic medications, intensive lifestyle intervention, and weight reduction strategies^①②^ Monitoring: More frequent multidimensional assessment (virological parameters, metabolic status, fibrosis evaluation, and HCC surveillance) may be considered^③^; shorter intervals for imaging-based HCC surveillance may be considered in high-risk individuals^②^

Management strategies within this framework are categorized into three evidence-based groups, indicated by ①②③ in the table:.

^①^Guideline-supported interventions: Standardized interventions recommended in current CHB or MASLD guidelines.

^②^Clinically supported strategies: Approaches supported by observational clinical evidence, including cohort studies and meta-analyses.

^③^Exploratory integrative strategies: Strategies proposed through integration of current mechanistic studies and clinical observations regarding virus–metabolism–immune interactions. These approaches have not yet been directly validated in large randomized controlled trials specifically involving populations with CHB with MASLD and are intended to provide a conceptual framework for clinical risk stratification rather than serve as independent diagnostic or therapeutic recommendations.

**Figure 2:**
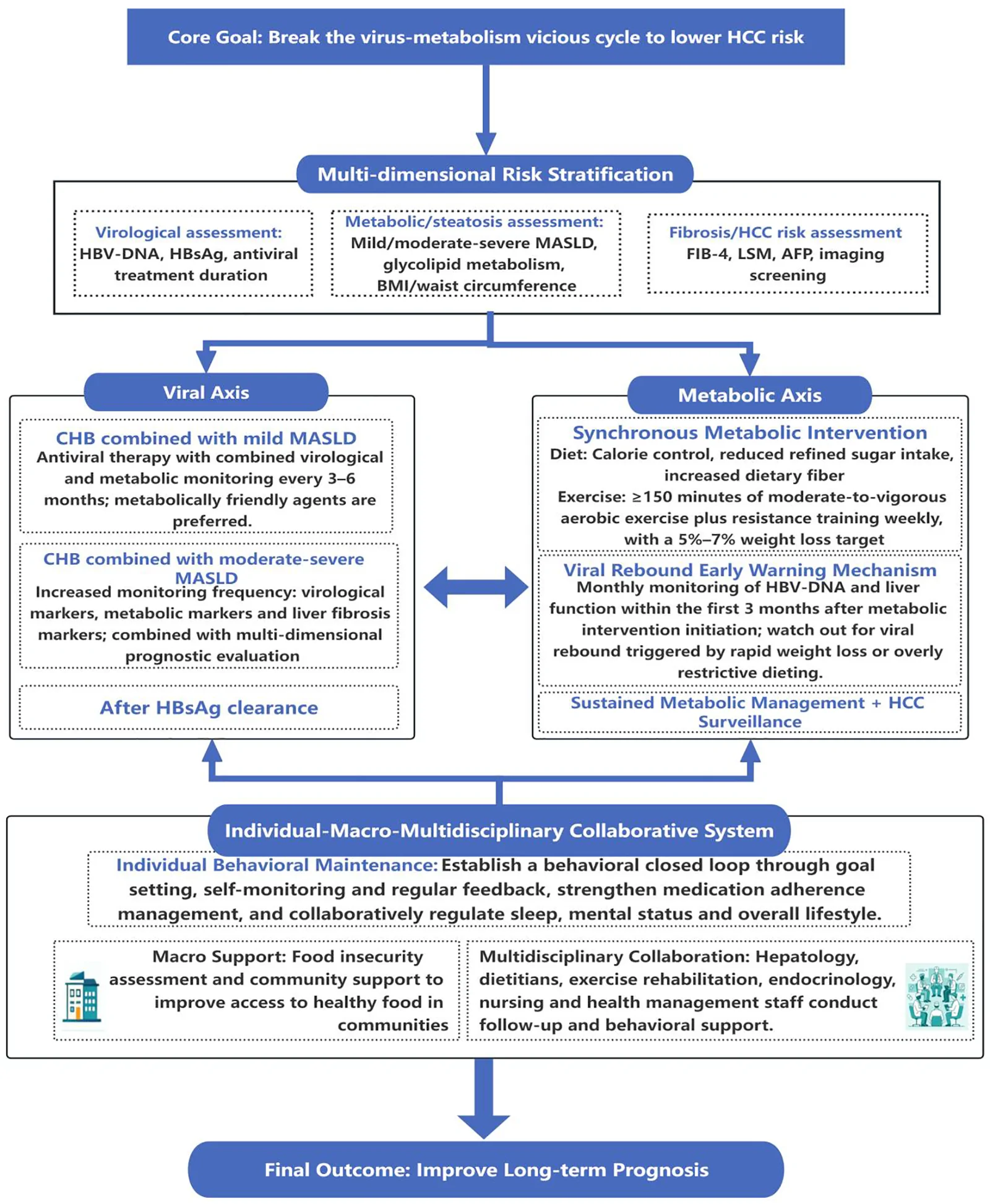
Conceptual clinical decision pathway based on the virus–metabolism dual-axis dynamic risk stratification framework.

Based on the above risk stratification framework, we further integrated and visualized the corresponding management strategies for each risk quadrant (**Figure 2**). It should be emphasized that this framework is not intended to replace existing CHB or MASLD guidelines, but rather represents an integrative approach built upon current evidence-based management principles for patients with CHB with MASLD. Interventions including antiviral therapy, metabolic risk factor management, and HCC surveillance are primarily based on current guideline recommendations. In contrast, dynamic risk adjustment, optimization of monitoring strategies, and dual-axis coordinated management considering the combined effects of viral activity and metabolic abnormalities represent exploratory integrative strategies derived from existing mechanistic studies and clinical evidence, which require further validation in prospective studies. Therefore, **Figure 2** is intended to illustrate a potential clinical decision-making approach based on virus–metabolism dual-axis risk stratification, rather than to establish a new clinical guideline.

#### 4.2.1 Viral axis: antiviral strategies and HCC surveillance under metabolic stratification

Based on the aforementioned virus–metabolism dual-axis risk stratification framework (**Table 3**), management of the viral axis in patients with CHB and MASLD should not be limited to HBV replication control alone, but may incorporate the degree of metabolic abnormalities into dynamic risk assessment, thereby forming an integrated management approach combining viral suppression, metabolic intervention, and HCC risk surveillance. For patients with CHB and mild MASLD, antiviral therapy can generally follow the principles recommended in current CHB management guidelines. Meanwhile, a virus–metabolism integrated monitoring approach may be considered according to individual metabolic status, including dynamic assessment of virological and hepatic parameters such as HBV DNA, HBsAg, HBeAg, ALT, and AST, together with metabolic indicators including body weight, waist circumference, lipid profiles, and glucose metabolism parameters. When hepatic steatosis progresses or metabolic abnormalities worsen, lifestyle interventions may be further intensified, with reassessment of liver fibrosis status and HCC risk (66).

For patients with CHB and moderate-to-severe MASLD, who frequently present with obesity, insulin resistance, dyslipidemia, and chronic low-grade inflammation, the risk of fibrosis progression and HCC development may be increased (6). Therefore, on the basis of standardized antiviral therapy, more comprehensive evaluation incorporating liver imaging, alpha-fetoprotein (AFP), and non-invasive fibrosis assessment may help identify individuals with elevated risks. For patients with substantial metabolic abnormalities, advanced fibrosis, or other HCC-related risk factors, intensified imaging surveillance and dynamic fibrosis assessment may be considered according to disease characteristics and clinical risk profiles. When selecting long-term antiviral strategies, individual metabolic conditions, including body weight, glucose and lipid metabolism, renal function, and bone metabolism, may also be taken into consideration while maintaining antiviral efficacy and a high genetic barrier to resistance, in order to minimize potential metabolic burdens during long-term treatment. Several retrospective studies have suggested that long-term antiviral therapy may be associated with an increased risk of metabolic abnormalities; however, the underlying mechanisms and causal relationships remain to be further clarified (24). Furthermore, patients with CHB and moderate-to-severe MASLD may exhibit improved virological or serological responses while maintaining an elevated risk of HCC. Therefore, clinical risk assessment should not rely solely on virological parameters such as HBV DNA suppression, ALT normalization, or HBsAg decline. Instead, a multidimensional assessment incorporating virological indicators, metabolic parameters, liver fibrosis markers, and imaging findings may provide a more comprehensive evaluation of long-term disease risk. Even after achieving HBsAg seroclearance, patients with persistent obesity, diabetes, dyslipidemia, or advanced liver fibrosis may still require continued long-term HCC surveillance and metabolic risk management.

#### 4.2.2 Lifestyle–pharmacological combined intervention and dynamic virological monitoring

Based on the aforementioned mechanisms underlying CHB with MASLD, metabolic intervention may not only improve hepatic steatosis and insulin resistance but may also influence disease progression through the modulation of lipotoxicity, inflammatory microenvironment, and immunometabolic status. Therefore, metabolic management may be incorporated into the overall therapeutic strategy at an early stage. In patients with CHB and MASLD, metabolic intervention may provide benefits beyond improving steatosis and insulin resistance, as it may also affect viral replication, hepatic inflammation, and long-term liver outcomes. Accordingly, metabolic management may be considered as an integral component of comprehensive disease management. Lifestyle modification remains the foundation of metabolic intervention. Dietary management is generally based on total energy restriction and optimization of dietary composition, including reduced intake of saturated fatty acids, trans fatty acids, fructose, and refined carbohydrates, with appropriate increases in high-quality protein, unsaturated fatty acids, and dietary fiber to reduce intrahepatic lipid accumulation and inflammatory responses (67). Dietary strategies may be further individualized according to patients’ dietary patterns, cultural backgrounds, and metabolic characteristics. Exercise interventions are generally centered on moderate-intensity aerobic activities, such as brisk walking, cycling, and swimming, with a cumulative duration of at least 150 minutes per week, combined with appropriate resistance training to improve insulin sensitivity, promote fat utilization, and preserve muscle mass (68).

Although lifestyle intervention represents a cornerstone of MASLD management, achieving and maintaining therapeutic targets remains challenging, and evidence supporting its independent effects on liver histology and long-term clinical outcomes remains limited. A recent multidisciplinary guideline indicated that improvements in dietary quality have moderate evidence levels for liver-related clinical outcomes (LoE 3), whereas the evidence supporting exercise benefits for liver histological improvement and fibrosis reduction remains relatively limited (LoE 5), with insufficient evidence among individuals with normal body weight and MASLD (65). Therefore, lifestyle intervention may need to be combined with pharmacological management of metabolic abnormalities.

Pharmacological treatment may follow a risk-stratified approach. For patients with CHB and MASLD accompanied by T2DM, dyslipidemia, or obesity, individualized treatment targets may be developed through multidisciplinary collaboration among hepatologists, endocrinologists, and cardiovascular specialists, referring to the EASL-EASD-EASO clinical practice recommendations (65). For patients with T2DM, glucose-lowering therapies with additional weight reduction and cardiovascular benefits, such as glucagon-like peptide-1 receptor agonists (GLP-1 RAs, e.g., semaglutide) and emerging metabolic-targeted agents such as dual receptor agonists, may represent potential therapeutic options (69, 70). Odium-glucose cotransporter 2 inhibitors (SGLT-2 inhibitors) may also provide metabolic benefits in selected patients (71). For patients with dyslipidemia, statin therapy may be considered after assessment of hepatic safety. For patients with obesity, anti-obesity medications may be incorporated when appropriate. Regarding liver-directed pharmacological approaches, available studies suggest that vitamin E may be considered in selected non-cirrhotic MASLD patients, while silymarin may exert anti-inflammatory and antioxidant effects, and ursodeoxycholic acid (UDCA) may potentially provide anti-inflammatory, anti-fibrotic, and metabolic benefits (72). Some studies have further suggested that UDCA may have lipid-lowering and cardiovascular protective effects (73), and expert opinions from the Polish Society of Cardiology have also proposed its potential role in reducing cardiovascular risk among patients with MASLD (74). Metabolic-targeted therapies may be more applicable during earlier stages of MASLD, whereas anti-fibrotic interventions may be more relevant for patients with advanced fibrosis or cirrhosis (75). It is noteworthy that even Resmetirom, an approved thyroid hormone receptor-β (THR-β) agonist, achieved histological response rates of only approximately 30% at 52 weeks, and data regarding its efficacy and safety in patients with CHB and MASLD remain unavailable (76). Furthermore, evidence regarding the long-term metabolic benefits of emerging pharmacological therapies remains insufficient, highlighting the need for future studies specifically targeting this comorbid population.

In addition, patients with CHB and MASLD may experience HBV reactivation or fluctuations in liver function during periods of rapid weight loss, strict low-calorie diets, or substantial short-term metabolic changes (77). Therefore, based on mechanistic considerations, an exploratory safety monitoring strategy may be considered. During the first three months after initiating metabolic intervention, increased monitoring frequency of virological parameters and liver function tests may be considered, particularly during periods of rapid metabolic changes, to facilitate early identification of potential viral rebound or worsening hepatic inflammation (78). Because metabolic fluctuations in patients with CHB and MASLD may be accompanied by changes in HBV activity, metabolic management in this population may require concurrent attention to HBV replication status and hepatic inflammatory responses. This represents an important distinction from the management of MASLD without underlying chronic HBV infection.

#### 4.2.3 Individual–macro level integrated multidisciplinary collaboration

Based on individual and macro-level regulatory factors, an integrated framework linking individual behavioral maintenance with broader environmental support may be considered. At the individual level, health behavior change theories can be incorporated to facilitate sustainable dietary management, regular physical activity, and medication adherence through goal setting, self-monitoring, and periodic feedback (79). or patients at risk of food insecurity, assessment of food accessibility may be necessary, and support from community health resources may be involved when appropriate. At the macro level, external determinants, including community food availability, socioeconomic conditions, and accessibility of health resources, may influence patients’ dietary behaviors and metabolic management. Enhancing community-based support may improve the sustainability of interventions and reduce metabolic risk exposure through upstream approaches (55). At the clinical level, current guidelines advocate the establishment of routine multidisciplinary collaboration models (80). Hepatologists are primarily responsible for antiviral treatment strategies and HCC surveillance; nutritionists and exercise specialists contribute to individualized metabolic interventions; nursing and health management professionals provide follow-up care and behavioral support; and public health professionals participate in the assessment of macro-level determinants and coordination of community resources. Through effective information sharing and interdisciplinary communication, these components may form a collaborative network integrating clinical care, behavioral intervention, and public health perspectives.

## 5 Conclusions and perspectives

CHB with MASLD is not simply an overlap of chronic viral infection and metabolic liver disease, but rather a complex disease state involving interactions among viral replication, metabolic dysregulation, immune-inflammatory responses, and host environmental factors. Therefore, its management may require a transition beyond single-disease treatment paradigms toward integrated strategies centered on comprehensive risk assessment and dynamic risk modulation. However, current evidence supporting the management of this comorbidity remains largely derived from single-disease studies and observational data, while direct interventional evidence specifically targeting patients with CHB and MASLD remains limited. High-quality prospective studies are needed to further evaluate the effectiveness and safety of current and emerging management strategies in this specific population. Future advances in comorbidity management are likely to focus on optimizing the coordination between viral control and metabolic intervention. On the one hand, emerging metabolic therapies, including THR-β agonists such as Resmetirom, GLP-1 receptor agonists, and other metabolic regulatory agents, have provided new therapeutic options for MASLD. However, their efficacy, safety, and potential effects on HBV-related disease progression in patients with CHB and MASLD require further investigation. On the other hand, novel HBV therapeutic strategies, including small interfering RNA (siRNA) and antisense oligonucleotides, have provided new possibilities for achieving functional cure. Nevertheless, whether these approaches can further reduce residual HCC risk among patients with CHB and MASLD requires confirmation through long-term follow-up studies. Furthermore, with the advancement of multi-omics technologies, future research may enable molecular classification and individualized risk prediction of patients with CHB and MASLD through integration of genomic, transcriptomic, and immune microenvironmental characteristics. Single-cell RNA sequencing and spatial transcriptomics may further elucidate dynamic interactions among viral factors, metabolic abnormalities, and immune cell networks. Artificial intelligence-based approaches may integrate virological parameters, metabolic indicators, imaging findings, and clinical characteristics to optimize risk stratification and long-term surveillance strategies. These research directions may provide further evidence supporting the proposed virus–metabolism dual-axis dynamic management framework and facilitate the transition of CHB with MASLD management from empirical intervention toward precision-based risk management.
